# Predictors of knowledge and practice of newborn care among post-natal mothers attending immunisation clinics in Southeast Nigeria

**DOI:** 10.4314/gmj.v56i3s.14

**Published:** 2022-09

**Authors:** Adaoha P Agu, Ifeyinwa C Akamike, Ijeoma N Okedo-Alex, Adanna A Umeokonkwo, Chidinma O Ogbonna-Igwenyi, Odinaka D Madumere, Chukwuemeka O Keke

**Affiliations:** 1 Department of Community Medicine, Alex Ekwueme Federal University Teaching Hospital, Abakaliki, Nigeria; 2 Department of Community Medicine, Ebonyi State University, Abakaliki, Nigeria; 3 African Institute for Health Policy and Health Systems, Ebonyi State University, Abakaliki, Nigeria; 4 Department of Paediatrics, Alex Ekwueme Federal University Teaching Hospital, Abakaliki

**Keywords:** newborn, neonatal mortality, post-natal care, evidence-based practice, Southeast Nigeria

## Abstract

**Objectives:**

Evidence-based newborn care practice recommended by WHO reduces neonatal mortality and improves neonatal outcomes. This study assessed the knowledge, practice-associated factors and predictors of essential newborn care among post-natal mothers in two primary health care centres in Southeast Nigeria.

**Methods:**

**Design:**

A cross-sectional total population study

**Setting:**

Two primary health care centres in two local government areas in Southeast Nigeria.

**Participants:**

Post-natal mothers who attended immunisation clinics

**Data collection:**

Quantitative data was collected over four weeks from 400 post-natal mothers. Chi-square test and logistic regression were carried out for associations and predictors, respectively. Analytical decisions were taken at p<0.05 and 95% confidence interval.

**Outcome measures:**

Knowledge, attitude, the practice of essential newborn care; predictors of practice

**Findings:**

Mean age of participants was 28.68±5.4. The majority (78.9%) had been counselled on newborn care and 85.2% delivered in a formal health facility. The majority (77%) had good knowledge of essential newborn care and practices (61%). More than half (62.3%) reported support from health workers. Predictors of good practice were older age (AOR: 0.435; 95%CI: 0.212–0.893), being married (AOR: 8.095; 95%CI: 3.732–17.558), living in the urban area (AOR: 0.478; 0.291–0.784), and having good knowledge of newborn care (AOR: 0.239; CI: 0.139–0.411).

**Conclusions:**

Good practice was identified in the majority. Being married, older, living in urban areas and having good knowledge were predictors of good practice. Health facility delivery, continuous support by health workers and post-natal education to mothers in rural areas are recommended policy priorities.

**Funding:**

None declared

## Introduction

The survival of newborns receives critical attention internationally. Despite progress in the reduction of neonatal mortality worldwide, sub-Saharan Africa bears the highest burden, with a neonatal mortality rate of 27 deaths per 1000 live births in 2019.^1^ Majority of newborn deaths occur in the first week of life, and^2^–^4^ scaling up cost-effective interventions in essential newborn care is crucial.^2^ Every Newborn Action Plan recognises the importance of strengthening health care for women and newborns as part of integrated health services for Reproductive, Maternal, Newborn, Child and Adolescent Health (RMNCAH).^5^

Essential newborn care involves early initiation of exclusive breastfeeding, prevention of hypothermia (delay bathing in the first 24 hours, skin-to-skin contact, hat), appropriate cord care, eye care, assessment for danger signs, counselling on danger signs and home care and routine immunisations. Close observation for 24 hours before discharge and post-natal contacts at least at days 3,6–7, and 6 weeks are recommended.^5^,^6^ Preterm neonates, those small for gestational age or with neonatal infections, are managed using guidelines.^5^ Introducing essential newborn care protocols early in hospitals improves essential newborn care practices.^7^

Furthermore, delayed initiation of breastfeeding increases the risk of neonatal mortality.^8^

Nigeria, with its consistently high neonatal mortality rate (37 deaths per 1000 live births in 2013^9^ and 36 deaths per 1000 live births in 2018,^2^ is far from the SDG target 3.2 of reduction of neonatal mortality to at least as low as 12 per 1000 live births by 2030. The Federal Government has made several attempts to reverse this trend. The integrated maternal, newborn and child health (IMNCH ) strategy revised in 2013 and the national “call to action” to end preventable newborn deaths in Nigeria in 2014 are examples.^10^ More recent is the revised National Reproductive Health Policy 2017, which aims to reduce maternal, perinatal, neonatal and child morbidity and mortality.^11^ There is paucity of published studies in Nigeria on the knowledge and coverage of a comprehensive package of essential newborn care practices among mothers within the first six weeks of childbirth. Globally, breast-feeding is the most widely reported of the essential new-born care practices prioritised by WHO but information on all the practices is important.^11^ Evidence from Nigeria, suggests suboptimal to moderate levels of knowledge and practice of essential newborn care.^12^,^13^ For example in one study, 59.3% practiced immediate skin to skin contact while only 10.1% initiated breastfeeding within 30 minutes of birth.^12^ In another study, poor knowledge of chlorhexidine gel for cord care in 62.8% of the mothers was found.^13^ Similarly, in a study of forty health facilities, no newborn received the full package of essential newborn care.^14^

In Ebonyi State, only 56.6% of women of reproductive age who had a live birth five years preceding the latest Demographic Health Survey delivered in a health facility; 58.3% had their deliveries attended by a skilled provider.^15^ There may be areas of conflict between recommended newborn care practices and local beliefs and practices, as was found in Ethiopia.^16^,^17^ This highlights the need for data on newborn care practices among women to build local evidence for targeted interventions to address the apparent implementation challenges of essential newborn care in Nigeria. We, therefore, undertook this study to determine the knowledge, attitude, practice, associated factors and predictors of essential newborn care among mothers attending immunisation clinics in Ebonyi State, Southeast Nigeria.

## Methods

### Study Setting

This study was carried out in Ebonyi State, Southeast Nigeria. It is inhabited and populated primarily by the Igbos. The city of Abakaliki is its capital and largest city. There are 13 local government areas (LGA) in the state. This study was carried out in two primary healthcare centres in two local government areas in the state: Izzi LGA, which has a total of 56 PHCs and Abakaliki LGA, which has a total of 29 PHCs.

### Study Design

The study was a descriptive cross-sectional study.

### Study Population

The study population was post-natal mothers who attended the immunisation clinics.

### Inclusion Criteria

This includes post-natal mothers who were present at data collection.

### Exclusion Criteria

Post-natal mothers who were present at the clinic but declined to answer the questionnaire.

### Sample Size Determination

The sample size was calculated using the formula (n = Z^2^PQ\d^2^)^18^ where n, is the minimum sample size, Zα is the standard normal deviate corresponding to a 2-sided level of significance of 5%, P is the proportion of the outcome of interest from a previous study or report, Q = 1-P, and d is the desired level of precision (usually at 5% for single proportions). The proportion of post-natal mothers with good knowledge of essential newborn care taken as 0.578^19^ was used to arrive at a minimum sample size of approximately 375. A total of 400 participants were selected to participate in the study.

### Sampling Technique

The two LGAs were purposively selected (Izzi-rural and Abakaliki-Urban). PHCs were purposively selected (one from each LGA) because of their high patient patronage. Proportionate allocation of the sample size was done based on the average number of post-natal mothers that attend the selected clinics monthly from clinic records. All eligible post-natal mothers were consecutively enrolled over four weeks.

### Study instruments and data collection

A self-administered, pre-tested semi-structured questionnaire adapted from previous studies was used to collect the data.^20^,^21^ Data collection was carried out by three research assistants and was carried out over four weeks. Questions asked related to the respondent's last baby.

### Measurement of variables

The independent variables include socio-demographic and other family characteristics such as age, marital status, level of education, employment status, residence, family type, and parity.

### The dependent variables include:

Eleven questions were used to assess the knowledge of women about essential newborn care. A correct answer for each question was scored 1 and 0 for a wrong answer.

The score varied from 0–11 points and was classified into two levels:

Poor knowledge: 0–6 scores (<55%)

Good knowledge: 7–11 scores (55–100%)

Nineteen 5-point Likert scale questions (with strongly disagreed scored as 1 point and strongly agreed as 5 points) were used to assess attitude with total scores ranging from 19–95. This was then classified into two categories:

Positive attitude: 48–95 scores (≥50%)

Negative attitude: 19–47 (<50%).

Twelve multiple-choice questions were used to assess the practice of essential newborn care. A correct answer for each question was given a score of 2, and 0 for a wrong answer. The score varied from 0–50 points and was classified into two levels:

Good practice: 25–50 scores (≥50%)

Poor practice: 0–24 scores (<50%)

The cut-off of 50% was based on Bloom's cut-off point, which categorises <50% as poor. This cut-off has been used in other studies.^22^ Feasibility and affordability were assessed by asking questions about the ease and cost of practising newborn care.

### Data analysis

Data were entered and analysed using SPSS (version 20) statistical software. Descriptive analysis was used to summarise data. The result was presented as frequency and percentages (for categorical variables), means and standard deviation (SD) for continuous variables. The Chi-square test of statistical significance was used to determine the association between socio-demographic variables and newborn care practice. Logistic regression was carried out to determine predictors of practice. Analytical decisions were taken at p<0.05 and 95% confidence interval.

### Ethical Consideration

Ethical approval for the study was obtained from the Health Research and Ethics Committee of Ebonyi State University, Abakaliki, Nigeria, EBSU/ DRIC/UREC/Vol.o5/081. Written informed consent was obtained. Respondents were assured of confidentiality and anonymity, and participation was voluntary.

## Results

Table 1 shows the socio-demographic characteristics of the respondents, with a mean age of 28.68 years and the majority falling between 25 to <35 years. Majority (87%) of them were married. Few (2.3%) of them do not have any formal education.

**Table 1:** Socio-demographic and obstetric characteristics of respondents

Variable	Frequency (%) n=400
**Age (mean ±SD)**	28.68±5.4
**Age range**	
**15-<25years**	94 (23.5)
**25-<35years**	253(63.3)
**35-<45years**	50 (12.5)
**45 and above years**	3 (0.8)
**Marital Status**	
**Single**	50(12.5)
**Married**	348 (87.0)
**Separated**	2 (0.5)
**Occupation**	
**Civil servant**	14 (3.5)
**Trader/Business**	157 (39.3)
**Tailor**	65 (16.3)
**Hairdresser**	45 (11.3)
**Farmer**	6 (1.5)
**Others**	81 (20.3)
**Unemployed**	32 (8.0)
**Level of Education**	
**No formal Education**	9 (2.3)
**Primary**	40 (10.0)
**Secondary**	225 (56.3)
**Tertiary**	126 (31.5)
**Residence**	
**Rural**	116 (29.0)
**Urban**	284 (71.0)
**Family Type**	
**Monogamous**	378 (94.5)
**Polygamous**	22 (5.5)
**Parity**	
**Primipara**	170 (42.5)
**Multipara**	230 (57.5)
**Place of Delivery**	
**Health facility**	340 (85.2)
**Home and TBA**	60 (14.8)
**Mean No of ANC vis-its** **during pregnancy**	5±2.02

Most live in urban areas, and there are more monogamous families, while over half (57.5%) were multiparous. The obstetric history of the respondents showed that 85% delivered in health facilities (primary, secondary and tertiary) and the mean number of ANC visits among the respondents was 5±2.02 . Primipara were 42.5%.

Table 2 shows that majority of the respondents knew about newborn care. Majority of the respondents (97%) knew that colostrum should be used to feed the baby. Most (76%) respondents knew of skin to skin care. A little above half of the respondents (58.8%) knew that sterilised scissors should be used to cut the cord and 35% knew that chlorhexidine should be applied to the cord after cutting. Most respondents (85.3%) knew that not feeding well in a newborn baby was a danger sign. All the respondents knew that convulsions and difficulty in breathing are not part of the signs of eye infection in a newborn baby. Overall, the majority of the respondents (77%) had good knowledge of ENC.

**Table 2:** Respondents' knowledge of essential newborn care

Variables	Frequency
**Knowledge about skin to skin care**	
**Yes**	304 (76.0)
**No**	96 (24.0)
**What is the correct time for the first bath**	
**Within 24 hours**	253(63.3)
**After 24 hours**	147(36.8)
**What instrument should be used to cut the cord**	
**Used blade**	4(1.0)
**New blade**	153(38.3)
**Unsterilised scissors**	8 (2.0)
**Sterilised Scissors**	235(58.8)
**What material should be applied to the cord**	
**Any type of oil**	15(3.8)
**Dettol**	1 (0.3)
**Spirit**	136 (34.0)
**Powder**	5(1.3)
**Toothpaste**	62 (15.5)
**Chlorhexidine**	140 (35.0)
**Cow dung**	1(0.3)
**Others**	40 (10.0)
**What is breast-feeding on demand**	
**When baby cries/looking for breast**	356 (89.0)
**When I feel like breastfeeding**	8 (2.0)
**Morning, afternoon, night food for baby**	31(7.8)
**Others**	5 (1.3)
**What is Exclusive breastfeeding (EBF)**	
**Breast milk + water**	23 (5.8)
**Breast milk only**	363 (90.8)
**Breast milk +formular**	14 (3.5)
**What is the correct duration of ( EBF)**	
**<6 months**	41(10.3)
**6 months**	351(87.8)
**>6 months**	8 (2.0)
**When should breast-feed be initiated**	
**Within one hour of birth**	347(86.8)
**After one hour**	53 (13.3)
**What should be done to colostrums**	
**Feed the baby with it**	388 (97.0)
**Throw it away**	12 (3.0)
Danger signs in a newborn baby **	
**Not feeding well**	341(85.3)
**Convulsions**	301(75.3)
**Drowsy or unconscious**	303(75.8)
**Stimulated or no movement at all**	251(62.8)
**Difficult/fast breathing**	270 (67.5)
**Raised temperature**	278 (69.5)
**Low body temperature**	228 (57.0)
**Bluish color**	164 (41.0)
**What are the vaccines given at birth**	
Polio (mouth drop)**	333 (83.3)
**Hepatitis (thigh)**	247 (61.8)
**BCG (upper arm)**	351(87.8)
What are the signs of eye infection in a baby**	
**Eye discharge**	299(78.8)
**Reddening of eye**	296 (74.0)
**Swollen Eye**	227 (56.8)
**Difficulty in breathing**	0 (0)
**Convulsion**	0(0)
**Overall Knowledge**	
**Good Knowledge**	308 (77.0)
**Poor Knowledge**	92 (23.0)

** multiple-choice questions

Table 3 shows the practice of newborn care. Most respondents (52.3%) gave the baby their first bath within 24 hours. None of the respondents applied cow dung to the cord, while 29.8% applied chlorhexidine. Few of the respondents (1.5%) breastfed three times a day for the first month, while most (94.3%) breastfed on demand. Most of the respondents (77%) vaccinated their babies at birth of which the majority (76.8%) was BCG vaccine. Most respondents (97%) have experienced drowsiness or unconsciousness as danger signs in their babies. Most respondents (61%) had good practices in newborn care.

**Table 3:** Respondents' practice of newborn care

Variables	Frequency (%)
**Did you ask for your baby to be placed skin to skin** **on your belly/chest after delivery?**	
**Yes**	187 (46.8)
**No**	213 (53.3)
**When did you give your baby the first bath?**	
**Within 24 hours**	209(52.3)
**After 24 hours**	191 (47.8)
**What instrument was used to cut the cord?**	
**Used blade**	3 (0.8)
**New blade**	175(43.8)
**Unsterilised scissors**	9 (2.3)
**Sterilised Scissors**	213 (53.3)
**What material do you apply to the cord?**	
**Any type of oil**	13 (3.3)
**Dettol**	6 (1.5)
**Spirit**	120 (30.0)
**Powder**	6 (1.5)
**Toothpaste**	91 (22.8)
**Chlorhexidine**	119 (29.8)
**Cow dung**	0(0)
**Others**	45 (11.3)
**Do you breast-feed on demand (when baby** **cries/looking for breast)?**	
**Yes**	377(94.3)
**No**	23 (5.8)
**How many times do you breastfeed daily in the** **first month of birth?**	
**3–6**	11(11.1)
**7–10**	74(104.1)
**>10**	15 (15.2)
**Do you/Are you practicing Exclusive breastfeeding?**	
**Yes**	305 (76.3)
**No**	95 (23.8)
**How long do you practice or intend to practice exclusive** ** breastfeeding?**	
**Less than 6 months**	137 (34.3)
**6 months**	238 (59.5)
**Greater than 6 months**	25 (6.3)
**When did you initiate breastfeeding?**	
**Within one hour of birth**	299 (74.8)
**After one hour**	101 (25.3)
**What did you do to the colostrum?**	
**Feed the baby with it**	374(93.5)
**Throw it away**	26 (6.5)
**Did you give any other food to the baby at birth** apart from drug?**	
**Yes**	87 (21.8)
**No**	313 (78.2)
If yes, which one/s?**	
**Honey/Sugar**	39 (9.8)
**Milk (other than breast milk)**	68 (17.0)
**Alcohol**	27(6.8)
**Water**	78 (19.5)
**Did you vaccinate your baby at birth?**	
**Yes**	308(77.0)
**No**	92(23.0)
**If yes to vaccinate baby at birth, which vaccines** were given?**	
**Polio (mouth drop)**	284 (71.0)
**Hepatitis (thigh)**	182 (45.5)
**BCG (upper arm)**	307 (76.8)
**Overall Practice**	
**Good**	244 (61.0)
**Poor**	156 (39.0)

### Attitude of respondents to essential newborn care

Almost all the respondents (98.5%) agree /strongly agree with hospital delivery being good for mother and child. Most of the respondents (84.5%) had a positive attitude towards newborn care.

### Respondents' perception of ease of practice, affordability of essential newborn care and support from health workers

Majority of the respondents (60.8%) found it easy /very easy to practice essential newborn care, and only 17.5% rated it expensive/very expensive. More than half (62.3%) reported receiving support from health workers.

Table 4 shows predictors of newborn care knowledge: marital status, employment status and residence. The married were three times more likely to have good knowledge (AOR: 3.378; 95%CI: 1.488–7.670). The unemployed were about 15 times more likely to have good knowledge (AOR: 15.774; 95%CI: 1.998–124.558), while those living in the rural area were three times more likely to have good knowledge (AOR: 3.125; 95%CI: 1.607–6.076).

**Table 4:** Predictors of Knowledge of newborn care

Variable	AOR	CI	P-value
**Age**		**Lower- Upper**	
**15–24 years**	0.808	0.319–2.045	0.65
**25–34 years**	1.528	0.705–3.311	0.28
**≥35years**	1		
**Marital Status**			
**Married**	3.378	1.488–7.670	<0.01*
**Others**	1		
**Level of Education**			
**Primary and below**	0.399	0.095–1.668	0.21
**Secondary and above**	1		
**Employment status**			
**Unemployed**	15.774	1.998–124.558	0.01*
**Employed**	1		
**Residence**			
**Rural**	3.125	1.607–6.076	<0.01*
**Urban**	1		

* statistically significant

Table 5 shows that knowledge and place of residence were predictors of good attitude. Those who live in the rural area were more likely to have good attitude (AOR: 6.943; 95%CI: 2.031–23.739). Those who had poor knowledge were less likely to have good attitude towards newborn care (AOR: 0.021; CI: 0.009–0.049).

**Table 5:** Predictors of attitude towards newborn care

Variable	AOR	CI	P-value
**Age**		**Lower- Upper**	
**15–24 years**	0.502	0.093–2.714	0.423
**25–34 years**	0.504	0.108–2.343	0.382
**≥35years**	1		
**Marital Status**			
**Married**	0.641	0.197–2.084	0.460
**Others**	1		
**Residence**			
**Rural**	6.943	2.031–23.739	<0.01*
**Urban**	1		
**Family Type**			
**Monogamous**	0.170	0.014–2.017	0.160
**Polygamous**	1		
**Knowledge**			
**Poor**	0.021	0.009–0.049	<0.01*
**Good**	1		

* statistically significant

Table 6 shows that age, marital status, knowledge and place of residence were predictors of good practice. The younger age groups were less likely to have good practice (AOR: 0.281; 95%CI: 0.116–0.683 for age group 15–25 years and AOR: 0.421; 95%CI: 0.195–0.910).

**Table 6:** Predictors of newborn care practice

Variable	AOR	CI (Lower	*P-value*
**Age**			
**15–24 years**	0.281	0.116–0.683	0.01*
**25–34 years**	0.421	0.195–0.910	0.03*
**≥35 years**	1		
**Marital Status**			
**Married**	4.701	2.032–10.874	<0.01*
**Others**	1		
**Residence**			
**Rural**	0.388	0.228–0.659	<0.01*
**Urban**	1		
**Parity**			
**Multiparous**	0.883	0.527–1.479	0.64
**Primiparous**	1		
**Knowledge**			
**Poor**	0.239	0.139–0.411	<0.01*
**Good**	1		

The married were more likely to have good practice (AOR: 4.701; 95%CI: 2.032–10.874), while those living in the rural area were less likely to have good practice (AOR: 0.388; 0.228–0.659). Poor knowledge was less likely to have good practice (AOR: 0.239; CI: 0.139–0.411).

## Discussion

This study assessed mothers' knowledge, attitude and practices towards newborn care, the associated factors and predictors of knowledge, attitude and practice. Additionally, their perception of the ease of practice, the affordability of the recommended practices and support from health workers were determined. It was carried out in primary health care centres, which are the health service delivery platforms for the majority of the populace. In a survey in Southeast Nigeria, newborns and immunisation services were the most available services across primary healthcare facilities.^23^

The majority of the respondents had good knowledge of skin-to-skin care, and this high proportion (76%) contrasts with findings in Ethiopia (43.9%).^24^ This result is similar to a study done in Bangladesh, where 70.6% of respondents knew about thermoregulation.^20^ The high proportion may be because many attended antenatal care, and 85% delivered in health facilities where they may have received education on newborn care practices.

Similarly, breastfeeding knowledge among mothers was high with most mothers aware of exclusive breastfeeding (EBF), breastfeeding initiation within one hour of birth, the importance of colostrum, and EBF until six months of age. This is comparable with findings in Ethiopia, where 87.8% of post-natal mothers had proper knowledge of EBF.^24^ The good knowledge of EBF could be because majority of our respondents had been counselled on newborn care and more than half were multiparous women so that they may have received this education repeatedly. In addition, majority of the mothers had at least secondary education, which could also have contributed to their good knowledge. This highlights the value of female education. Some knowledge questions, however, had a low proportion of respondents with good knowledge, including the correct time of the first bath and the correct material used for cord care. This poor knowledge could be due to the traditional practices which mothers usually teach their daughters during “Omugwo” (a practice in the south-eastern part of Nigeria where mothers visit daughters who had just given birth to care for baby and mother).

Most post-natal mothers knew all the danger signs (except for bluish discoloration) and the vaccines given at birth. This high level of knowledge can be explained by the high average number of ANC visits. Also, there were more respondents with male babies and mothers might seek to ensure that their male children are prevented from diseases considering the preference for male children in African culture. This may have contributed to their good knowledge about vaccines given at birth.

Overall, majority of the respondents had a positive attitude towards newborn care, affirming the importance of healthcare facility delivery (only 15% of respondents delivered outside a healthcare facility). This positive attitude towards newborn care is advantageous because it is a first step to behaviour change. The importance of continuous health education of women about newborn care during antenatal visits and immunisation clinic visits is inferred from our findings. It sustains the gains recorded in the fight to reduce neonatal deaths. Interestingly, less than one third of the respondents supported the non-practice of mixed feeding (25.8% disagreed/strongly disagreed that mixed feeding should not be practised) despite their good knowledge of EBF and positive attitude. This could be because most women believe that it's strange not to give a baby any other food apart from breast milk; some find it hard to believe that breast milk contains the water and every nutrient that a baby below six months of age requires. Further studies may be required to probe the reasons for this belief. In addition, we recommend that health workers pay attention to providing the little details about exclusive breastfeeding when educating mothers.

The overall good practice of newborn care among post-natal mothers found in this study (61%) is comparable to Nepal's (66.2%) findings.^25^ Concerning the practice of skin to skin placement, less than half of the women practiced it, despite the relative high level of knowledge. This is lower than a recent study in Nigeria (59.3%)^12^ but higher than another study in Nigeria(14.6%)^14^ and North Ethiopia (25.8% ).^16^ About cord care, for the greater majority of the mothers, sterilised scissors were used to cut the cord. However, very few (29.8%) applied chlorhexidine, although Nigeria adopted the use of chlorhexidine for cord care in 2016 in line with the recommendation of WHO on the use of chlorhexidine for cord care for home births in areas where neonatal mortality rate is above 30/1000 live births.^26^ Fortunately, the application of cow dung to the cut stump was reportedly not practiced. This is a big win, though further education on the continued use of sterilised scissors and chlorhexidine is needed. WHO and UNICEF recommend initiation of breastfeeding within one hour of birth, exclusive breastfeeding for the first 6 months of the infant and breastfeeding on demand.^27^ Majority of the mothers initiated breastfeeding within the first hour of birth, fed their babies with colostrum, and breastfed their newborns on demand.

The high proportion of respondents (74.8%) who practiced early initiation of breastfeeding differs from findings in Nigeria (44%)^14^, (10.1%);^12^ Pakistan (48%)^28^ and Bangladesh (40%).^29^ However, our findings were reflected in a study in Ethiopia^24^, which revealed that the majority (89.8%) of participants practised early breast-feeding initiation. In the study in Pakistan,^28^ 43% of respondents discarded the colostrum, unlike our figure of 6.5%. A possible explanation may be that 78.9% of our respondents were counselled on newborn care practices, and 62.3% were supported in practice by health workers. Early initiation of breastfeeding would be taught by health workers considering the long history of the Baby Friendly Hospital Initiative (BFHI) in Nigeria.^30^ The high proportion of practising early breastfeeding initiation is encouraging because delayed initiation increases the risk of neonatal mortality. Water was the most common added to feed for those who practised mixed feeding in alignment with published and anecdotal evidence in Nigeria. The NDHS 2018^15^ revealed that only 29% of mothers practice exclusive breastfeeding in Nigeria. It is worth exploring if the health workers themselves giving the health education believe that the newborn can thrive without added water. Concerning vaccination at birth, good practice was established for polio and BCG vaccines. The good practice of newborn care is expected since majority of the mothers had good knowledge and positive attitude towards vaccination. However, the proportion (61%) of respondents with overall good practice is suboptimal. This underscores the need for continuous health education on newborn care. Further studies on the details provided by health workers to mothers are needed to ensure they are properly educated.

Over half of the respondents agreed that ENC was easy to practice, sustainable, and affordable. This is advantageous in improving newborn care practices because women will more likely stick with practices that will cost them little or nothing to achieve optimum results. The majority was supported by health workers in several ways, especially through health education during ANC/immunisation visits. Continuous support and education by health workers should be encouraged to improve newborn care practice further.

Being married, older age, having good knowledge of ENC and living in the urban area were predictors of good practice of newborn care. Being married and older will increase the likelihood of exposure to peer or other support groups positively influencing behaviour. Contrary to expectation, we did not find a significant association between the education of the mother and good practice of essential newborn care (therefore regression analysis was not done for education) unlike some studies,^12^,^29^,^31^ although in some of these studies, the association test was for specific components of the practice domain which was not carried out here. In accord with our results, a study in Ethiopia did not demonstrate education as a predictor of good practice of ENC.^32^

marital status is a predictor of both good knowledge and good practice. Gender inequalities limit women's access to RMNCAH services in Ebonyi State.^33^ This may be more pronounced in situations where the unmarried undergo childbirth, because of our societal norms. Therefore, it is not unexpected that the married were more likely to know and practice essential newborn care. However, this result needs to be interpreted cautiously since 87% of our respondents were married. Living in rural areas was a predictor of knowledge and practice but in different directions. Those living in rural area were less likely to have good practice. Our results are similar to that of a systematic review of ENC utilisation in Ethiopia where it was observed that urban residence was strongly associated with appropriate ENC utilisation.^34^ The authors of that review proffer better health service accessibility in the urban area as one possible reason.

The finding on rural residents less likely to have good practice is unexpected since numerous donor partners are working in partnership with the government in Ebonyi State, which have a strong focus in rural areas where majority of the populace live and where health service utilisation indices are usually worse than urban areas. On the other hand, our finding of rural residents more likely to have good knowledge is in line with the rural focus of many of these partners. Perhaps sufficient efforts have not been made to translate the knowledge of ENC into practice in their activities. However other factors may be at play and further investigation is needed to understand the disparity.

The predictors of good attitude were knowledge and residence. Knowledge was a predictor of both attitude and practice, with good knowledge being more likely to result in good attitude and good practice. This is not surprising. Newborn care practices are influenced by traditional perceptions often passed on from mothers, grandmothers and elderly women in the community.^35^ Good knowledge and understanding of appropriate practices should overcome these perceptions. A study in Lesotho demonstrated that good knowledge was significantly associated with exclusive breastfeeding in the first six months.^36^ Also, in a study in Ethiopia, mothers who received four and more antenatal follow up visits were more likely to have good newborn care practices than those who received one; attributed to the possibility of increased knowledge of the components and importance of newborn care practices received during ANC.^37^

Knowledge has also been shown to influence positively the acceptability of newborn care health initiatives.^35^

Limitations of this study were that practice was self- reported and no observation of these mothers was done. However, their responses were encouraged to be as sincere as possible. Since the study was conducted in only two primary health centres, the generalisability of the findings may be limited. Additionally, the women's responses may have been subject to recall bias.

## Conclusion

In this study, there was overall good knowledge, positive attitude and good practice of essential newborn care among the women. However, the practice of chlorhexidine for cord care was reported in less than a third of the respondents. Residence and knowledge were predictors of attitude while age, marital status, residence and knowledge were predictors of good practice. Good knowledge of essential newborn care provided at antenatal care and delivery in health facilities is important for its acceptability and practice among post-natal mothers.

Health workers' healthcare delivery, continuous support, and health education to mothers in the antenatal and post-natal period are recommended policy priorities to improve and sustain good practice.
